# Transcription start site profiling of 15 anatomical regions of the *Macaca mulatta* central nervous system

**DOI:** 10.1038/sdata.2017.163

**Published:** 2017-10-31

**Authors:** Margherita Francescatto, Marina Lizio, Ingrid Philippens, Luba M. Pardo, Ronald Bontrop, Mizuho Sakai, Shoko Watanabe, Masayoshi Itoh, Akira Hasegawa, Timo Lassmann, Jessica Severin, Jayson Harshbarger, Imad Abugessaisa, Takeya Kasukawa, Piero Carninci, Yoshihide Hayashizaki, Alistair R. R. Forrest, Hideya Kawaji, Patrizia Rizzu, Peter Heutink

**Affiliations:** 1 Italian Institute of Technology, Department of Neuroscience and Brain Technologies, Via Morego 30, Genova 16163, Italy; 2 RIKEN Center for Life Science Technologies, Division of Genomic Technologies, 1-7-22 Suehiro-cho, Tsurumi, Yokohama, Kanagawa 230-0045, Japan; 3 RIKEN Yokohama Institute, Omics Science Center, 1-7-22 Suehiro-cho, Tsurumi, Yokohama, Kanagawa 230-0045, Japan; 4 Biomedical Primate Research Centre, Postbox 3306, Rijswijk 2280 GH, The Netherlands; 5 Department of Dermatology, Erasmus MC Cancer Institute, Burg. s' Jacobplein 51, Rotterdam 3015 CA, The Netherlands; 6 RIKEN Preventive Medicine and Diagnosis Innovation Program, 1-7-22 Suehiro-cho, Tsurumi, Yokohama, Kanagawa 230-0045, Japan; 7 Telethon Kids Institute, The University of Western Australia, 100 Roberts Road, Subiaco, Western Australia 6008, Australia; 8 Harry Perkins Institute of Medical Research, 6 Verdun St, Nedlands, Western Australia 6009, Australia; 9 RIKEN Advanced Center for Computing and Communication, Preventive Medicine and Applied Genomics Unit, 1-7-22 Suehiro-cho, Tsurumi, Yokohama, Kanagawa 230-0045, Japan; 10 German Center for Neurodegenerative Diseases, Otfried-Müller Straße 23, Tübingen 72076, Germany

**Keywords:** RNA sequencing, Gene expression profiling, Gene expression, Transcriptomics

## Abstract

Rhesus macaque was the second non-human primate whose genome has been fully sequenced and is one of the most used model organisms to study human biology and disease, thanks to the close evolutionary relationship between the two species. But compared to human, where several previously unknown RNAs have been uncovered, the macaque transcriptome is less studied. Publicly available RNA expression resources for macaque are limited, even for brain, which is highly relevant to study human cognitive abilities. In an effort to complement those resources, FANTOM5 profiled 15 distinct anatomical regions of the aged macaque central nervous system using Cap Analysis of Gene Expression, a high-resolution, annotation-independent technology that allows monitoring of transcription initiation events with high accuracy. We identified 25,869 CAGE peaks, representing bona fide promoters. For each peak we provide detailed annotation, expanding the landscape of ‘known’ macaque genes, and we show concrete examples on how to use the resulting data. We believe this data represents a useful resource to understand the central nervous system in macaque.

## Background and Summary

*Macaca mulatta*, also known as rhesus macaque and named hereafter macaque for simplicity, is a small non-human primate whose natural habitat includes a large portion of India, Pakistan and China^1^. Because of its small size and remarkable adaptability, and its high evolutionary relatedness to humans, the macaque is as an important model organism in biomedical sciences^2^ as mouse. After chimpanzee, it was the second non-human primate whose genome has been fully sequenced. A first macaque assembly was released in 2007, and comparisons with human and chimpanzee genomes gave initial insight into primates evolutionary relationships^3^. In 2015 the Baylor College of Medicine submitted a novel assembly^4^, doubling the contigs size thanks to the assembling of longer fragments, and fixing several assembly and annotation errors of the previous version.

Despite an improved resolution at the genome level, the macaque transcriptome has not benefited of the same level of analyses performed in human, where noncoding RNAs (including enhancer-derived RNAs), alternative transcription initiation, and RNA isoforms were uncovered^5–7^. While it is particularly relevant to study all these newly discovered transcript types in the context of evolution, the current annotation in macaque does not allow us to examine this aspect in depth. For example, for the latest rheMac8 genome build there are currently 6,460 Reference Sequence (RefSeq) annotated genes, against the 66,466 available for the recently released human hg38 build available from UCSC Genome Browser^8^. Similarly, the latest Ensembl gene build for macaque, although more comprehensive, consists of 56,748 transcript models in contrast to the 199,234 ones for human. Even though predictions from a variety of algorithms can provide an initial assessment of the genes present in a given organism, observation of RNA molecules is necessary to validate the predictions. Moreover, the expression of genes is often specific to cells or tissues^9^, and alternative promoter usage associated to distinct transcript isoforms has an impact on the overall expression and its regulation^10,11^, which can be relevant in diseases, including those of the brain.

The FANTOM (functional annotation of the mammalian genome) consortium is an international collaboration established in Japan in the year 2000 aiming to unveil all the functional elements in mammalian genomes paired with reliable annotations^12–14^. FANTOM5, the fifth iteration of the collaboration, revealed the most comprehensive promoter and enhancer atlases for human and mouse by using Cap Analysis of Gene Expression (CAGE) combined with single molecule sequencing^5,7,15,16^. The CAGE technique is a well-established expression profiling approach that allows to identify novel or previously unannotated/mis-annotated gene starts by sequencing 5′-end of RNAs transcribed by RNA polymerase II, as well as to quantify their expression levels^17^. Thanks to the high precision of CAGE signal^18^ it is therefore possible to characterize transcription start sites (TSS) and promoter regions of genes.

**Figure 1:**
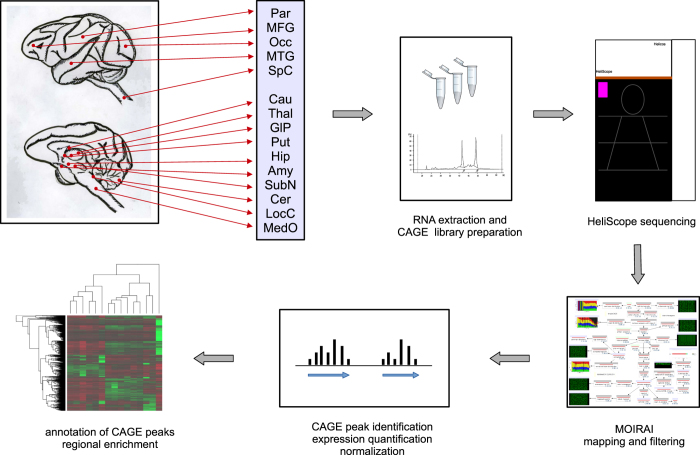
Experimental design of this study. The figure shows a schematic workflow from sampling of CNS anatomical regions to subsequent sample and data processing. The full sample names corresponding to the abbreviations used in the figure are reported in Table 1.

Here we present CAGE-based expression profiles for 15 regions of the macaque central nervous system (CNS), obtained within the context of FANTOM5 (Table 1). A flow of the analysis is visualized in Fig. 1. Overall, we identify 25,869 CAGE peaks, representing bona fide transcription start sites (TSS) of genes expressed in the macaque CNS. Considering the gene models available for the rheMac8 assembly only (Table 2), 71.2% of the peaks is located within 500 bp of an annotated TSS. Overall, 80.4% of the peaks are associated to a gene when including gene models from human lifted over to macaque (also listed in Table 2). We also investigate sequence features around macaque TSSs. We observe that 14,877 CAGE peaks (57.5%) overlap a CpG island while 1,219 of them (4.7%) harbor a TATA-box site within a 70 bp region around their 5′-end. We also find that 3,471 peaks (13.4%) overlap a repetitive element, with simple repeats and LINE/L1 being the most represented classes. We perform several analyses to confirm that the expression profiles we obtained are of high quality. First and foremost, we observe a generally high correlation between samples, despite the fact that the libraries represent distinct anatomical brain regions. In addition, our expression profiles show agreement with results from Peng *et al.*^19^ and Bakken *et al.*^20^.

This is, to date, the most detailed CNS dataset on macaque obtained using an annotation-independent transcription profiling technique consisting of experimentally defined promoters with detailed annotation and expression values. Given the availability of ‘matching’ CAGE data from human (*Homo sapiens*) in FANTOM5 (ref. 7), these data can be useful to understand CNS functions and complexity from an evolutionary perspective, rather than representing a general mapping of transcriptome in macaque. Both the macaque and the human CAGE profiles were obtained from samples extracted from aged donors and could be used to study processes in the aged primate brain or late onset brain disorders, including neurodegenerative diseases. In addition, we provide concrete examples on how to use the data presented.

## Methods

### Sample collection and RNA extraction

The dissection of all regions was performed from fresh brain sections. Tissue was stored at −80 °C until further processing. Total RNA was extracted from frozen tissues using the Trizol tissue kit according to instructions provided by the manufacturer (Invitrogen) and further purified with RNeasy mini kit (Qiagen). RNA quality was assessed using the RNA Integrity Number (RIN) with the Agilent RNA Nano kit using the Agilent Bioanalyzer. All the samples used, their RIN values and library IDs are listed in Table 1.

### Library preparation and sequencing

For each sample one CAGE library was prepared using a protocol adapted for single molecule sequencing as previously described^15^. The libraries were subsequently sequenced on HeliScope sequencer^16^ following manufacturer’s instructions.

### Mapping and data processing

Sequenced libraries were first filtered for reads aligning to ribosomal DNA (with up to two mismatches) by rRNAdust^7^. The remaining reads were aligned to rheMac8 reference genome using the probabilistic mapping tool delve (see Code Availability).

Data mapping workflow was implemented in Moirai, a system that allows creation and customization of sequencing data processing pipelines^21^.

Expression quantification was performed as previously described^7^. In short, 1-base pair TSSs were first obtained from genome alignments and then the number of aligned reads to the same 5′ position was used to quantify their expression levels. We further normalized the values, as done previously^7^, in order to account for sample specific variations and library sizes. We kept only those reads with 99% alignment accuracy (mapping quality q>20) and sequence identity above 85% for downstream analyses. Mapped reads and single base frequencies of transcription initiation activities (TSS) are available via DDBJ (see Data Records, Data Citation 1).

### Identification of CAGE peaks

CAGE peaks, which represent promoters of active genes, were defined using the Decomposition Peak Identification (DPI) method, previously described^7^ (see Code Availability). Briefly, the method performs decomposition of the CAGE signal across a set of samples and subsequently clusters the signal at TSSs, by considering a minimum distance and expression level thresholds. For each peak, normalized tags per million (TPM) were computed using the relative log expression method implemented in edgeR^22^.

### Comparison with publicly available annotation datasets

Peaks were annotated using all gene models available for download from UCSC genome Browser^8^ for the rheMc8 genome build, such as Augustus^23^ and Genescan^24^ predictions, Ensembl genes^25^ and experimentally validated RefSeq^26^ and Expressed Sequence Tags (ESTs). To increase the coverage of the annotated peaks, we additionally considered human RefSeq and GENCODEv24 (ref. 27) (comprehensive annotation) models, also downloaded from UCSC genome browser. Human RefSeq and GENCODE models were lifted from hg38 to rheMac8 using UCSC liftOver tool (available from http://hgdownload.soe.ucsc.edu/admin/exe/), with default parameters. We considered a CAGE peak to be associated to a given gene model if it was located within a (−500, +500) region centered at the feature start, as in previous studies^7^. Annotation files were created using a combination of ‘intersectBed’ and ‘groupBy’ commands from the bedTools suite^28^ (version 2.17.0). The total numbers of CAGE peaks annotated for each model are provided in Table 2 while complete annotation information for each peak is reported in Data Citation 2.

### Identification of region specific CAGE peaks

To identify CAGE peaks enriched in any given region we calculated relative expressions for all peaks. Given a CAGE peak P, its relative expression in a region was calculated as P_rel_=log_10_((1+P_region_)/(1+P_median_)), with P_region_=normalized expression of peak P in the current region and P_median_=median normalized expression of P across all regions. For each peak a relative expression above 1 indicates that it is preferentially expressed in that region. The full list of regionally enriched macaque CAGE peaks is provided in Data Citation 3, along with corresponding gene symbols (based on macaque RefSeq, macaque Ensembl and human RefSeq genes lifted over to macaque).

### Comparison with published human CAGE expression profiles

The FANTOM5 human promoter set includes normalized expression data for all 15 corresponding macaque brain regions (Data Citations 4 and 5). We extracted CAGE peaks coordinates and their expression for the matching samples from the human set, all derived from the same donor, and used them as a reference to compare sequence composition and overlap with repetitive elements, general expression profiles and regionally enriched peaks.

The dataset consists of 74,649 robust CAGE peaks, after filtering out very lowly expressed peaks, for expression comparison. Specifically, we required a CAGE peak to be expressed above 1 TPM in at least one sample. The samples and their relative quality metrics are provided in Table 1. We point out that, compared to the CAGE peaks set in macaque, the corresponding human set is larger, as the identification of peaks in that case was performed on ~1,000 samples across multiple cell and tissue types. At a 10 TPM threshold, the number of CAGE peaks in both species is comparable (~1.4 fold difference), with 14,437 peaks for macaque and 20,762 for human.

### Additional processing

Overlaps with repeat elements and CpG islands downloaded from UCSC for both macaque (rheMac8) and human (hg19), were computed using bedtools^28^. Enrichment of TATA binding protein (TBP) motifs across the CAGE peaks was obtained using HOMER motif discovery software^29^ (see Code availability). In particular, the function ‘annotatePeaks.pl’ was employed to identify the CAGE peaks harboring a TATA-box motif in a region (−500, +200) around the peak. The PWM used for the TATA-box enrichment analysis corresponds to the POL012.1 motif from Jaspar^30^, the default HOMER motif database. The annotation function calculates the distance of a motif from the center of the target sequences. This needs to be taken into account when interpreting the resulting enrichments.

Correlations, normalization of expression values, enriched expression calculation and plots were performed using R version 3.1.2 (http://www.R-project.org/).

### Ethics statement

The study has been approved by the ethical committee (DEC) of the Biomedical Primate Research Centre, Lange Kleiweg 139, 2288 GJ Rijswijk, The Netherlands. Study DEC No. 606.

### Code availability

Mapping was performed using delve, a probabilistic aligner specifically developed for handling data derived from HeliScope single molecule sequencing. The software is available at fantom.gsc.riken.jp/5/suppl/delve/.

The DPI peak calling was performed using our own software. It can be freely downloaded at this URL (https://github.com/hkawaji/dpi1/).

HOMER motif analysis suite is available at http://homer.salk.edu/homer/.

## Data Records

The data resulting from sequencing (fastq format), mapping (bam format) and TSS profiling (bed format) of macaque samples is deposited in the DNA Data Bank of Japan (Data Citation 1). The data is also available for direct download from FANTOM5 data repository (http://fantom.gsc.riken.jp/5/datafiles/phase2.2/). The data resulting from sequencing (fastq format), mapping (bam format) and TSS profiling (bed format) of human samples is deposited in the DNA Data Bank of Japan (Data Citations 4 and 5). Macrodissection microarray normalized expression profiles from multiple regions of the macaque CNS were downloaded from the NIH Blueprint Non-Human Primate (NHP) Atlas (http://www.blueprintnhpatlas.org/static/download). Normalized RNAseq expression profiles from multiple tissues and multiple non-human primate species were downloaded from Nonhuman Primate Reference Transcriptome Resource (http://nhprtr.org/phase2.html).

## Technical Validation

### RNA quality and mapped reads

To assess the quality of the starting material, a RIN (RNA Integrity Number) score was determined for each of the samples (Table 1). The RIN is a standard measurement evaluating the integrity of the RNA quality in a sample. Low RIN values indicate higher levels of degradation, suggesting that the sample should not be used to prepare CAGE libraries. The RIN numbers of our samples are all above 7.7, satisfying the minimum threshold recommended for library preparation in case of CAGE libraries (RIN=7)^31^. We report, in addition, the number of mapped tags per library, which varies from 3.0 to 6.2 millions, with an average of 4.5. The total number of mapped tags can be influenced by many factors, such as RNA degradation, or low initial RNA amount, other than completeness of a genome assembly. As done previously^7^, we required a minimum of 0.5 M mapped tags per sample. All 15 samples were well above this threshold, with a median of 4.5 million mapped reads and median mapping rate of 50.9% (Table 1). To verify the coverage of the currently available genome by CAGE, we measured the mapping rate, defined as the ratio between all single mapped reads and all filtered extracted reads (that is, sequences remaining after artifacts and ribosomal DNA removal). We observe that the average mapping rate for macaque libraries is 53.6%, higher than the average obtained for the matching human libraries (40.4%, Table 1).

### Correlation between expression profiles across libraries

**Figure 2:**
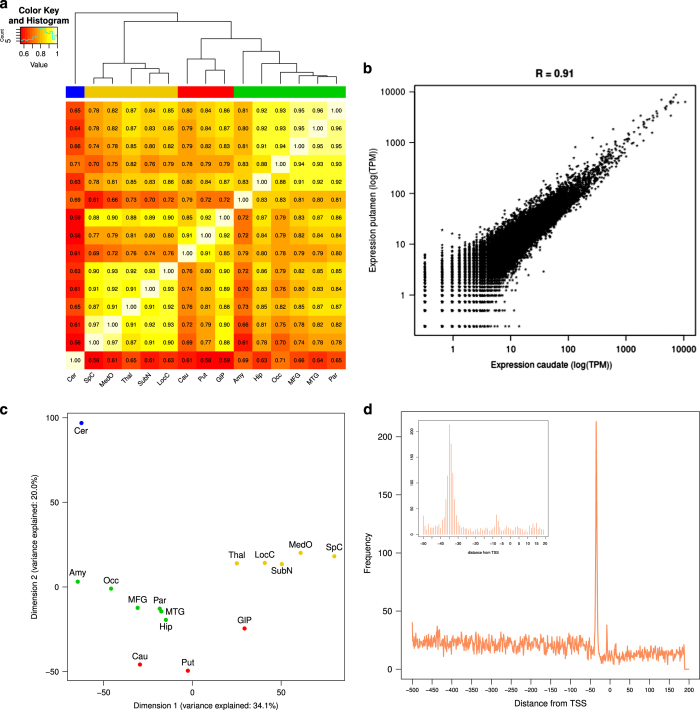
Promoter features and quality assessment of CAGE data. (**a**) Graphical representation of the Spearman correlation values between all 15 macaque expression profiles presented in this study. The top dendrogram shows that the samples cluster in four major groups, as highlighted by the colored bar above the heatmap. (**b**) Scatterplot and Spearman correlation value for a pair of regions showing a high degree of similarity (caudate and putamen). Axes represent log-transformed TPM expression. (**c**) Multi-dimensional scaling representation of the samples included in the study, color-coded to show the clustering of samples (colors corresponding to those in **a**). (**d**) Frequency profile showing TATA-box enrichment upstream of the promoter. Insert shows a zoom-in view of the region (−50, +20), with a clear enrichment around 35 bases upstream of the TSS.

We calculated the Spearman correlation between the normalized expression profiles identified (represented as a heatmap in Fig. 2a). As reflected by the clustering, indicated by the column dendrogram, values are particularly high between structurally and functionally related regions of the CNS. For instance, the correlation between caudate and putamen (Fig. 2b), which share a similar neuronal composition together with the dorsal striatum, is 0.91. Correlation is 0.93 between substantia nigra and locus coeruleus and 0.94 between cortical samples (frontal, temporal, parietal and occipital gyruses). Similarly, high correlation values between structurally and functionally related regions are observed across human expression profiles (Table 3).

### Multidimensional scaling of expression profiles reflects functionally related regions

In Fig. 2c we show a multi-dimensional scaling (MDS) representation of the samples described in this study. A clear separation of the cerebellum from all the other samples can be observed, as already suggested by correlation analysis, and previously reported in human as well^32^. This likely reflects both the particular cellular composition, which is distinctive with respect to other CNS districts^33^, and the rapid evolution of cerebellum in great apes and humans^34^. It is possible to distinguish three additional clusters of data points (color-coded in Fig. 2c). A first one (depicted in green) includes the cortex samples plus amygdala and hippocampus. The cortical regions make the external layer of the brain, and share functions related to the individual’s awareness (cognition and sensory), so their clustering together may reflect that. The presence of hippocampus and amygdala in this group can be also explained by functional and developmental relationships (all evolved from the primitive brain). A second cluster, depicted in red, consists of globus pallidus, caudate and putamen. The latter two are both part of the striatum and are involved in motor and learning activities. The remaining cluster, in gold, is more heterogeneous and includes samples anatomically belonging to the brain stem and basal ganglia. We also note the position of the globus pallidus, midway between the red and the gold clusters, perhaps reflecting direct contact (physically and functionally) with putamen and at the same time direct targeting of substantia nigra^35^.

### Promoter features and sequence composition

We investigated sequence composition around the TSSs by calculating the number of CAGE peaks identified in macaque that are located within a CpG island. Since CAGE peaks precisely locate TSSs of actively transcribed genes, we treat the terms CAGE peaks and TSSs as synonyms. CpG islands are computationally predicted regions of high GC di-nucleotides frequency that tend to be found at promoters^36^. For comparison, we performed the same analysis on CAGE peaks defined for a matching set of CNS regions profiled in human with the same technique (see Methods). We observe an overall higher proportion of CAGE peaks overlapping CpG islands in macaque with respect to human (57.5 versus 48.8%), even though the CpG island density in the two species is very close^37^ (Table 4). Next, we screened the CAGE peaks in macaque and in the corresponding human CNS samples for the presence of a TBP motif^38^. We observed clear enrichment for the TATA-box motif at around 35 bases upstream of the macaque TSS, in agreement with previous reports on human promoters^13^ (Fig. 2d). We note that the distances reported by HOMER (see Methods) are calculated between the center of the TSS and the center of the motif; we also note that the median length of the peaks with a TATA motif is 13 bp, therefore our findings are consistent with what is known to be the preferred TATA-box position (between −28 and −31 bp)^13^. The percentage of promoter regions containing a TATA motif within a (−50, +20) window is very similar between the human and macaque species (Table 4).

Since it was described in both human and mouse that an important portion of CAGE-defined TSS are located within repetitive elements^39^, we also investigated this property. We observe that 3,571 CAGE peaks (13.8%) overlap a repetitive element, with simple repeats and LINE/L1 elements being the most represented classes (peak annotation with respect to repeats, CpG and TATA-box is provided in Data Citation 6). The proportion of CAGE peaks overlapping repeat elements is overall lower than what we observe in our reference human dataset (19.6%).

### Association with annotated genes

Another way to assess the quality of our promoter dataset is comparing it with established gene models, as it is expected that most of the measured signal reflects the expression of known genes. This is particularly true for CAGE data, as the protocol is designed to specifically target regions of transcription initiation^17^. We downloaded 5 distinct macaque gene models from UCSC (Ensembl, RefSeq, Augustus, ESTs, Genscan) and used them to annotate the CAGE peaks identified (Data Citation 2). With the manually curated genes from RefSeq, only 24% of the CAGE peaks we identified could be annotated. By using the gene build provided by Ensembl, 55.6% of the peaks were annotated, which is higher than the rate of gene-associated CAGE peaks in human (82,150/184,827; 44% (ref. 7)). We assume that because the number of macaque CAGE libraries is much smaller than those profiled in human, the resulting peaks, which are calculated over the entire sets of samples for each species, capture mainly highly expressed promoters. By missing the rare promoters in macaque, the gene-peak association rate increases. When considering human hg38 RefSeq gene or GENCODEv24 genes projected to macaque (see Methods), 60.2 and 72% respectively of the CAGE peaks were annotated. Overall, a large proportion of the CAGE peaks identified (80.4%) results to be annotated, with differences depending on the reference chosen. A summary of the number of peaks annotated with respect to each of the gene models is provided in Table 2. In Data Citation 2 we provide the full annotation information for each CAGE peak.

### Regionally enriched CAGE peaks

**Figure 3:**
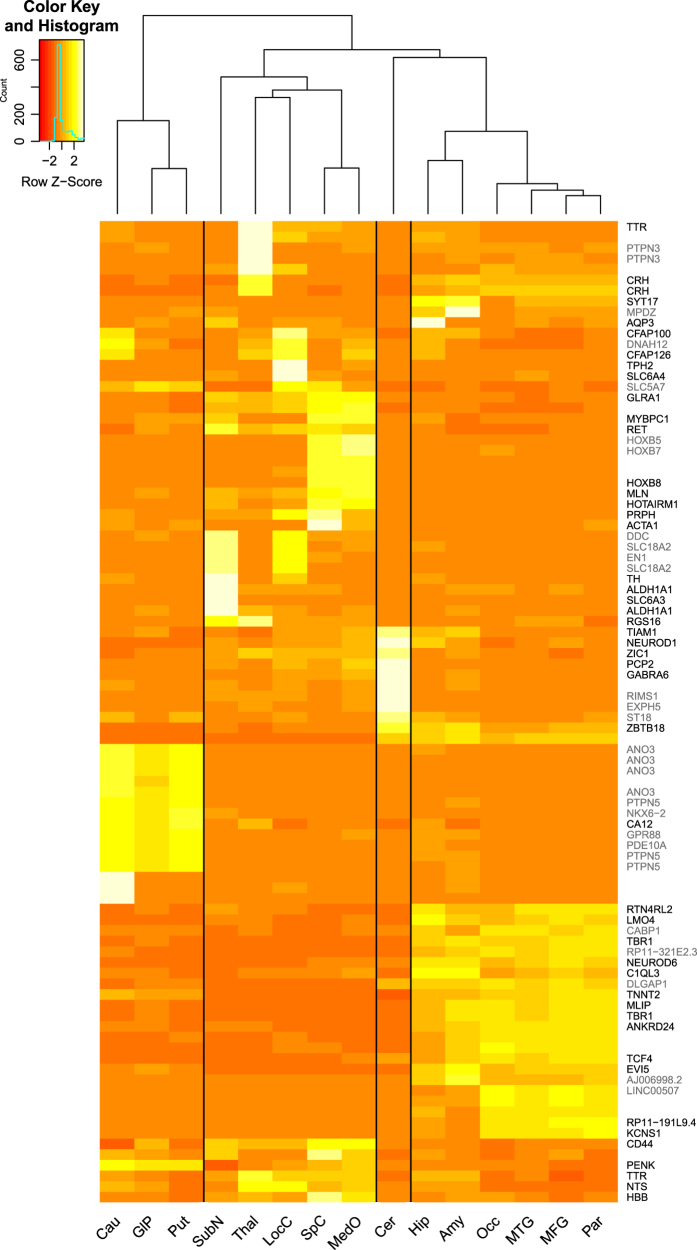
Expression patterns and annotation of regionally enriched peaks. The heatmap shows the TPM normalized expression profiles of the top 10 enriched CAGE peaks in each region. The black vertical lines highlight the separation of clustered regions as induced by the expression profiles (top dendrogram). Labels on the right side report the gene symbols (lifted over to rheMac8) corresponding to each peak, when such association is available. Black labels indicate symbols available in the native macaque annotations (either RefSeq or Ensembl), gray labels indicate symbols from human annotations (either RefSeq or GENCODE) lifted over to macaque.

To assess the resolution of our data in terms of region-specific signal, we extracted a list of enriched CAGE peaks for each region (see Methods). This resulted in 877 enriched peaks (corresponding to 642 unique peaks), which we provide in Data Citation 3 with the corresponding gene symbols for rheMac8 Ensembl and RefSeq, and projected hg38 RefSeq models. We then created a heatmap of region-specific expression patterns using the TPM normalized expression profiles (scaled in order to render the differences in expression easily identifiable) of the corresponding top 10 peaks enriched in each region (Fig. 3). Gene names are displayed for all those peaks that could be annotated, in black are all those available from macaque annotations and in gray those assigned thanks to the lift over of human annotations. As some of the samples are biologically and/or functionally extremely close, most of the peaks are specific for a subset of regions rather than for a single region. For example frontal, temporal, occipital and parietal gyruses share the majority of the enriched CAGE peaks, as shown in bright yellow in the heatmap, with the Potassium Voltage-Gated Channel Modifier Subfamily S Member 1 (*KCNS1*) showing the highest enrichment in all four regions (score>1.5, Data Citation 3). The most notable exception is given by cerebellum, for which most of the enriched genes are truly specific (97.0%). These include Purkinje Cell Protein 2 (*PCP2*), Cerebellin 1 and Cerebellin 3 precursors (*CBLN1*, *CBLN3*), all reported to be highly expressed in this region^40–42^.

It is important to notice that the choice of gene models can have a significant impact on the results: considering experimentally validated models only, such as rheMac8 RefSeq gene annotations, would lead us to conclude that the vast majority of the region-enriched CAGE peaks is un-annotated (only 11.1% of the peaks would be assigned a gene symbol). However, a larger number of annotations can be rescued when considering the rheMac8 Ensembl gene set (35.8% annotated), or the more comprehensive hg38 RefSeq genes lifted over to macaque (43.3% annotated). For example, the cerebellum-enriched genes Gamma-Aminobutyric Acid Type A Receptor Alpha6 Subunit (*GABRA6*), Regulating Synaptic Membrane Exocytosis 1 (*RIMS1*) and Neuronal Differentiation 1 (*NEUROD1*) are not found in the macaque RefSeq set, but are present in both the Ensembl and human RefSeq sets. So, in general, leveraging the human annotations lifted over to macaque allows us to assign a gene to a larger number of peaks, an example of which is the possibility to include more gene symbols as done in Fig. 3, giving us a tool to improve gene accuracy.

## Usage Notes

### Data exploration using the FANTOM5 portal

An online website is available (http://fantom.gsc.riken.jp) where all data generated within the FANTOM5 project are collected, and visualization and browsing tools are publicly accessible. Users interested in downloading the whole genomic coordinates of CAGE TSSs, peaks, expression values and gene associations for macaque as well as other organisms, such as human, can access our ftp site fantom.gsc.riken.jp/5/datafiles/latest/.

Moreover, the expression profile for a sample, or even the expression levels for a single gene across samples, can be visualized, together with dynamic statistics and data pooling operations, via our original ZENBU genome browser^43^ (http://fantom.gsc.riken.jp/zenbu/). A tabular version of the expression values for a subset of genes and/or for a subset of samples from the FANTOM5 collection can be obtained from the Table Extraction Tool TET (http://fantom.gsc.riken.jp/5/tet/).

An example demonstration of how to navigate our resource, and how to find out more about the data, is given below.

**Figure 4:**
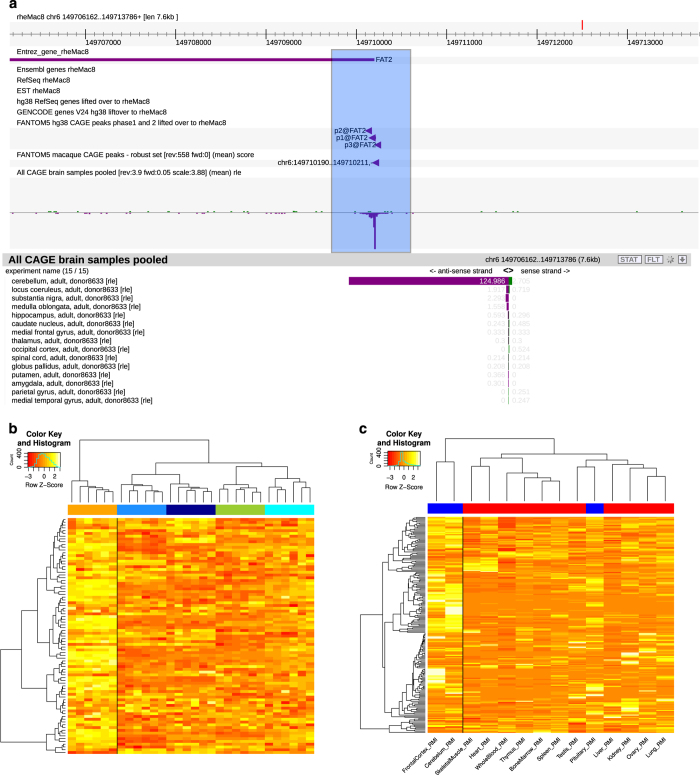
Visual exploration of the dataset presented and comparison with external data. (**a**) Screenshot from ZENBU, one of the resources available to explore the expression data presented in this study, showing one example of missing gene annotation. (**b**) Example of a comparison between CAGE and NIH Blueprint NPH Atlas data. The colors on the top bar label distinct macroscopical anatomical regions (orange=basal ganglia, dodger blue=occipital cortex, dark blue=medial frontal cortex, yellow green=hippocampal cortex, cyan=amygdaloid complex). (**c**) Example of a comparison between CAGE and NHPRTR datasets. The top bar distinguishes CNS (blue) and non-CNS (red) samples.

The semantic browser SSTAR^44^ stores information about samples and/or CAGE peaks. A human cerebellum sample page (http://fantom.gsc.riken.jp/5/sstar/FF:10166-103B4) lists all information and analysis results associated to that sample. Among the most enriched genes in cerebellum is *NEUROD1* (p1@NEUROD1), a known neuronal transcription factor involved in nervous system development and insulin regulation. We can compare the human *NEUROD1* CAGE peak (or better the peak associated to *NEUROD1*) to the corresponding macaque CAGE peak by determining, for example, whether this gene is also enriched in macaque. Indeed, among the top 10 enriched peaks in macaque cerebellum we find one that according to Ensembl gene annotation is associated to *NEUROD1* (CAGE peak ID ‘chr12:68735984..68735999,-’ in Data Citation 3). Conversely, the precision of CAGE in finding TSSs can help to resolve cases of ambiguous or unreported genes; for instance, the macaque cerebellum-enriched CAGE peak ‘chr6:149710190..149710211,-’ seems to have no association to known genes although (a) it is located at the 5′-end of the *FAT2* Entrez gene locus, (b) there is a cluster of lifted over human promoters to the *FAT2* gene (Fig. 4a), and (c) *FAT2* gene search in SSTAR browser shows it is highly enriched in human cerebellum (http://fantom.gsc.riken.jp/5/sstar/EntrezGene:2196), all points suggesting the possibility of this peak being a promoter for a *FAT2* gene in macaque. Similarly, another top enriched CAGE peak in macaque (chr4:70244748..70244762,+) is reported to have no associated RefSeq or Ensembl genes. This peak is located in an intron of the annotated macaque *RIMS1*, a gene involved in enhanced cognitive abilities in humans^45^, shows nearly exclusive expression in cerebellum, and matches the liftOver of human p5@RIMS1 promoter, almost certainly representing a promoter for a shorter *RIMS1* gene isoform in macaque (http://fantom.gsc.riken.jp/zenbu/gLyphs/#config=TJD69JeXM5ylAJTRKiJ45D;loc=rheMac8::chr4:70244705..70244804+). A search in SSTAR semantic browser for *RIMS1* gene in human confirms expression enrichment in cerebellum as well (http://fantom.gsc.riken.jp/5/sstar/EntrezGene:22999), specifically for p5@RIMS1 promoter (http://fantom.gsc.riken.jp/5/sstar/FFCP_PHASE2:Hg19::chr6:72922590..72922605,%2B). Proof of the fact that several un-annotated peaks represent the promoter of a gene can also be ascertained by exploring the dedicated configuration for macaque in ZENBU (http://fantom.gsc.riken.jp/zenbu/gLyphs/#config=TJD69JeXM5ylAJTRKiJ45D).

These examples also expose the dangers of using currently available, incomplete gene models, and show that gene models annotation can benefit from a technology such as CAGE.

### Comparison with other publicly available primate CNS expression datasets

The thorough annotation we provide for the expression profiles presented in this study makes it easy to perform comparisons with external datasets. As an example, we considered two recently published expression atlases including macaque samples corresponding to several regions of the CNS (see Data Records). We extracted normalized expression data of basal ganglia published from NIH Blueprint NHP Atlas^20^ (Data Records) for donors of 12 and 48 months of age and compared the expression signature between this dataset and ours. We selected the CAGE peaks enriched (see Methods) in the corresponding regions profiled (caudate, putamen, globus pallidus and substantia nigra). By matching gene symbols from our annotation with those reported in the study, we extracted the corresponding expression profiles. Figure 4b shows a heatmap for these expression profiles in which the subgroup of basal ganglia samples is well discriminated (samples labeled in orange). In a similar fashion, we obtained normalized expression data, relatively to the subset corresponding to macaque, from the non-human primate reference transcriptome resource^19^ (Data Records). We again selected all CAGE peaks regionally enriched and we extracted the corresponding NHPRTR expression profiles based on matched gene symbols. Figure 4c shows a heatmap of such profiles, with CNS samples labeled in blue and non-CNS samples in red. We note that while frontal cortex and cerebellum profiles are well separated from non-CNS samples, the pituitary gland, although being part of the CNS, falls within non-CNS tissues, as a hormone-secreting gland.

In conclusion, we describe a new and important resource to help understand central nervous system in macaque that can be compared with similar resources in humans and other species such as mouse.

## Additional information

**How to cite this article:** Francescatto, M. *et al.* Transcription start site profiling of 15 anatomical regions of the *Macaca mulatta* central nervous system. *Sci. Data* 4:170163 doi: 10.1038/sdata.2017.163 (2017).

**Publisher’s note:** Springer Nature remains neutral with regard to jurisdictional claims in published maps and institutional affiliations.

## Supplementary Material



## Figures and Tables

**Table 1:** List of the macaque and matching human samples (15 for each species) included in this study, with corresponding library ID, tissue name and basic QC values (RIN, total number of mapped reads and mapping rate).

**Library ID**	**Species**	**Tissue**	**Abbreviation**	**RIN**	**Mapped reads**	**Mapping rate (%)**
CNhs14182	Rhesus macaque	Amygdala	Amy	9	4,565,587	50.9
CNhs14174	Rhesus macaque	Caudate	Cau	8.4	3,348,210	44.68
CNhs14176	Rhesus macaque	Cerebellum	Cer	9.2	5,682,996	65.99
CNhs14171	Rhesus macaque	Globus Pallidus	GlP	8.1	4,486,692	50.57
CNhs14180	Rhesus macaque	Hippocampus	Hip	8.7	3,017,562	41.6
CNhs14172	Rhesus macaque	Locus Coeruleus	LocC	8.4	4,140,956	49.5
CNhs14177	Rhesus macaque	Medial Frontal Gyrus	MFG	8.1	5,576,066	65.76
CNhs14179	Rhesus macaque	Medial Temporal Gyrus	MTG	8.2	3,968,759	47.6
CNhs14175	Rhesus macaque	Medulla Oblongata	MedO	8.2	5,407,111	61
CNhs14169	Rhesus macaque	Occipital Gyrus	Occ	8.6	5,269,914	61.3
CNhs14181	Rhesus macaque	Parietal Gyrus	Par	8.6	3,883,216	48.8
CNhs14178	Rhesus macaque	Putamen	Put	8.1	4,493,463	52.28
CNhs14168	Rhesus macaque	Spinal Cord	SpC	7.7	4,282,060	51.33
CNhs14173	Rhesus macaque	Substantia Nigra	SubN	7.7	3,167,510	40.84
CNhs14170	Rhesus macaque	Thalamus	Thal	8.4	6,211,376	71.69
CNhs12311	Homo sapiens	Amygdala	Amy	6.9	14,101,506	41.5
CNhs12321	Homo sapiens	Caudate	Cau	7.2	15,501,176	44.06
CNhs12323	Homo sapiens	Cerebellum	Cer	6.9	17,383,592	44.66
CNhs12319	Homo sapiens	Globus Pallidus	GlP	6.8	14,100,137	40.64
CNhs12312	Homo sapiens	Hippocampus	Hip	6.7	15,910,618	41.21
CNhs12322	Homo sapiens	Locus Coeruleus	LocC	7.2	14,226,392	40.88
CNhs12310	Homo sapiens	Medial Frontal Gyrus	MFG	7.2	16,201,206	43.34
CNhs12316	Homo sapiens	Medial Temporal Gyrus	MTG	7	16,329,604	40.89
CNhs12315	Homo sapiens	Medulla Oblongata	MedO	7.5	15,199,808	43.08
CNhs12320	Homo sapiens	Occipital Gyrus	Occ	8.4	15,966,482	39.88
CNhs12317	Homo sapiens	Parietal Gyrus	Par	7.1	10,980,255	33.08
CNhs13912	Homo sapiens	Putamen	Put	8.3	2,262,558	27.58
CNhs12227	Homo sapiens	Spinal Cord	SpC	7.3	5,957,420	39.95
CNhs12318	Homo sapiens	Substantia Nigra	SubN	8.1	12,393,047	41.45
CNhs12314	Homo sapiens	Thalamus	Thal	7.3	18,287,224	43.51
We additionally report in this table the abbreviations used in the figures for each CNS anatomical region.						

**Table 2:** Number of CAGE peaks within 500 bp of at least one annotated gene model.

**rheMac8 gene model**	**Number of peaks**	**Percentage of peaks**
Augustus	11,435	44.2
EST	6,514	25.2
Genscan	5,953	23
refGene	6,248	24.1
Ensembl Gene	14,376	55.6
hg38 refGene liftOver	15,562	60.2
hg38 GENCODE liftOver	18,635	72

**Table 3:** Spearman correlation across human expression profiles derived from a matching set of CNS regions.

	**Amy**	**Cau**	**Cer**	**GlP**	**Hip**	**LocC**	**MFG**	**MTG**	**MedO**	**Occ**	**Par**	**Put**	**SpC**	**SubN**	**Thal**
**Amy**	1	0.87	0.72	0.81	0.92	0.83	0.9	0.9	0.79	0.88	0.88	0.65	0.74	0.73	0.83
**Cau**		1	0.69	0.79	0.85	0.8	0.84	0.83	0.76	0.81	0.82	0.69	0.7	0.68	0.82
**Cer**			1	0.67	0.72	0.7	0.73	0.73	0.73	0.73	0.72	0.52	0.64	0.62	0.68
**GlP**				1	0.84	0.89	0.8	0.81	0.86	0.83	0.77	0.65	0.85	0.9	0.93
**Hip**					1	0.86	0.89	0.9	0.83	0.89	0.88	0.64	0.77	0.76	0.86
**LocC**						1	0.82	0.82	0.89	0.83	0.8	0.63	0.84	0.86	0.89
**MFG**							1	0.94	0.78	0.92	0.93	0.64	0.72	0.71	0.82
**MTG**								1	0.78	0.93	0.93	0.64	0.72	0.71	0.83
**MedO**									1	0.79	0.76	0.59	0.85	0.85	0.87
**Occ**										1	0.91	0.64	0.75	0.75	0.84
**Par**											1	0.63	0.7	0.68	0.8
**Put**												1	0.58	0.58	0.66
**SpC**													1	0.86	0.84
**SubN**														1	0.89
**Thal**															1

**Table 4:** Proportions (as %) of CAGE peaks associated to TATA-box and CpG islands in macaque and human. For TATA, a region 70 bp around the TSS was used.

	**CpG+,TATA+**	**CpG+,TATA−**	**CpG−,TATA+**	**CpG−,TATA−**
**macaque**	2.1	55.4	2.7	39.8
**human**	1.5	47.3	2.7	48.6
