# Author Correction: Transcription start site profiling of 15 anatomical regions of the *Macaca mulatta* central nervous system

**DOI:** 10.1038/s41597-018-0003-4

**Published:** 2018-12-11

**Authors:** Margherita Francescatto, Marina Lizio, Ingrid Philippens, Luba M. Pardo, Ronald Bontrop, Mizuho Sakai, Shoko Watanabe, Masayoshi Itoh, Akira Hasegawa, Timo Lassmann, Jessica Severin, Jayson Harshbarger, Imad Abugessaisa, Takeya Kasukawa, Piero Carninci, Yoshihide Hayashizaki, Alistair R. R. Forrest, Hideya Kawaji, Patrizia Rizzu, Peter Heutink

**Affiliations:** 1 Italian Institute of Technology, Department of Neuroscience and Brain Technologies, Via Morego 30, Genova, 16163 Italy; 2 RIKEN Center for Life Science Technologies, Division of Genomic Technologies, 1-7-22 Suehiro-cho, Tsurumi, Yokohama, Kanagawa 230-0045 Japan; 3 RIKEN Yokohama Institute, Omics Science Center, 1-7-22 Suehiro-cho, Tsurumi, Yokohama, Kanagawa 230-0045 Japan; 4 Biomedical Primate Research Centre, Postbox 3306, Rijswijk, 2280 GH The Netherlands; 5 Department of Dermatology, Erasmus MC Cancer Institute, Burg. s’ Jacobplein 51, 3015 CA Rotterdam, The Netherlands; 6 RIKEN Preventive Medicine and Diagnosis Innovation Program, 1-7-22 Suehiro-cho, Tsurumi, Yokohama, Kanagawa 230-0045 Japan; 7 Telethon Kids Institute, The University of Western Australia, 100 Roberts Road, Subiaco, Western Australia 6008 Australia; 8 Harry Perkins Institute of Medical Research, 6 Verdun St, Nedlands, Western Australia, 6009 Australia; 9 RIKEN Advanced Center for Computing and Communication, Preventive Medicine and Applied Genomics Unit, 1-7-22 Suehiro-cho, Tsurumi, Yokohama, Kanagawa 230-0045 Japan; 10 German Center for Neurodegenerative Diseases, Otfried-Müller Straße 23, Tübingen, 72076 Germany

Correction to: *Scientific Data*, 10.1038/sdata.2017.163, Published online 31 October 2017

The authors regret that Luba M. Pardo was omitted in error from the author list of the original version of this Data Descriptor. This omission has now been corrected in the HTML and PDF versions.

The authors also regret that Anemieke Rozemuller was omitted in error from the Acknowledgements of the original version of this Data Descriptor. This omission has now been corrected in the HTML and PDF versions.

